# *In situ* production of a two-dimensional molybdenum disulfide/graphene hybrid nanosheet anode for lithium-ion batteries†

**DOI:** 10.1039/d0ra01503b

**Published:** 2020-04-14

**Authors:** Srikanth Mateti, Md Mokhlesur Rahman, Pavel Cizek, Ying Chen

**Affiliations:** Institute for Frontier Materials, Geelong Campus, Deakin University Waurn Ponds 3216 Victoria Australia m.rahman@deakin.edu.au ian.chen@deakin.edu.au

## Abstract

A solvent-free, low-cost, high-yield and scalable single-step ball milling process is developed to construct 2D MoS_2_/graphene hybrid electrodes for lithium-ion batteries. Electron microscopy investigation reveals that the obtained hybrid electrodes consist of numerous nanosheets of MoS_2_ and graphene which are randomly distributed. The MoS_2_/graphene hybrid anodes exhibit excellent cycling stability with high reversible capacities (442 mA h g^−1^ for MoS_2_/graphene (40 h); 553 mA h g^−1^ for MoS_2_/graphene (20 h); 342 mA h g^−1^ for MoS_2_/graphene (10 h)) at a high current rate of 250 mA g^−1^ after 100 cycles, whereas the pristine MoS_2_ electrode shows huge capacity fading with a retention of 37 mA h g^−1^ at 250 mA g^−1^ current after 100 cycles. The incorporation of graphene into MoS_2_ has an extraordinary effect on its electrochemical performance. This work emphasises the importance of the construction of the 2D MoS_2_/graphene hybrid structure to prevent capacity fading issues with the MoS_2_ anode in lithium-ion batteries.

## Abstract 2

A solvent-free, low-cost, high-yield and scalable single-step ball milling process is developed to construct 2D MoS_2_/graphene hybrid electrodes for lithium-ion batteries.

## Graphical abstract

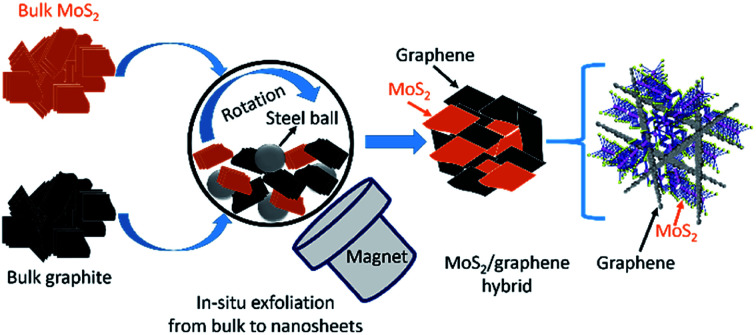

## Introduction

Electrode materials are key ingredients of any battery and the anode is a major component in lithium-ion batteries due to its electrochemical performance. Although, graphite is the most popular commercial anode material for lithium-ion batteries (LIBs), however, graphite-based anodes are unable to meet the market demands of high energy density due to the low theoretical capacity of 372 mA h g^−1^.^1^ It is, therefore, necessary to develop alternative anode materials with high capacity and excellent cycling stability. Recently, layer-structured molybdenum disulfide (MoS_2_) has attracted much attention due to its low cost, environmental friendliness, easy fabrication, and wide application in many fields, particularly rechargeable batteries.^2^ Even though MoS_2_ has potential to be used as an anode in LIBs with a high theoretical capacity of 670 mA h g^−1^, low electronic conductivity and large volume variations during the insertion and extraction of ions leads to poor cycling stability of the cells.^2,3^ To circumvent these problems, the hybridization concept is widely used, and electrochemical performance, especially cycling stability of the MoS_2_ anode is improved significantly. Meanwhile, carbons or other conductive materials have been integrated to form composites/hybrids with different geometries and dimensions, which could lead to improved lithium-ion storage performances in terms of both capacity and cyclic stability.^4–7^

**Fig. 1:**
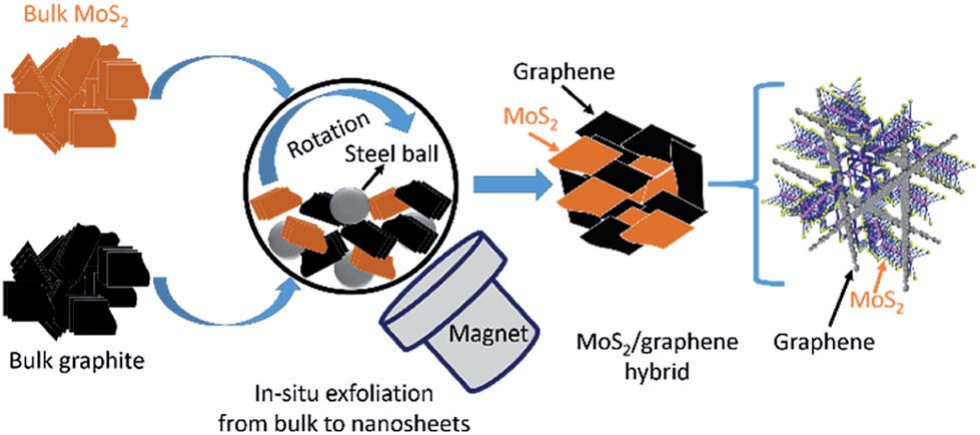
Schematic of *in situ* production procedure of MoS_2_/graphene hybrid nanosheets.

In general, a common problem in preparing MoS_2_ based electrodes is that most of the production methods produce small quantity (milligrams), which is drastically different from existing industrial processes. The materials production process is vital because, if the production technique could not be adopted by industry, it would not be possible to commercially implement the electrode materials no matter how wonderful they are. Herein, we develop a simple scalable ball-milling technique to produce a large quantity of MoS_2_/graphene hybrid nanosheet electrodes *via* an *in situ* generation of graphene and 2D MoS_2_ where bulk graphite and bulk MoS_2_ used as starting materials as presented in Fig. 1. The obtained structure of MoS_2_/graphene heterointerface is expected to enhance the electronic conductivity and store more Li^+^, thus results in better electrochemical properties. The electrochemical performance of the MoS_2_/graphene hybrid nanosheets is improved drastically over the single system of either MoS_2_ or graphite when used as an anode material in LIBs.

## Experimental

To prepare MoS_2_/graphene hybrid nanosheets, commercial bulk MoS_2_ (Sigma Aldrich-particle size ∼ 6 μm) and commercial bulk graphite (Sigma Aldrich-particle size < 20 μm) were used as starting materials. 4 grams of powder mixture with 2 grams of MoS_2_ and 2 grams of commercial graphite (weight ratio = 1 : 1) were loaded in a stainless steel milling container. Four hardened steel balls with a diameter of 25.4 mm each were also loaded to the same steel milling container where powder to ball ratio was maintained 1 : 1 (4 grams : 4 balls). The milling container was evacuated and purged with argon gas for several times and filled with anhydrous ammonia (NH_3_). Anhydrous NH_3_ is selected in our experiment because NH_3_ environment protects the original structure of materials while exfoliation to nanosheets.^8^ Both materials were milled in a rolling ball-mill at a rotation speed of 150 rpm for 10, 20, and 40 h at room temperature under an atmospheric pressure of 300 kPa. The obtained products were denoted as MoS_2_-G (10 h), MoS_2_-G (20 h), and MoS_2_-G (40 h), respectively. X-ray diffraction (XRD) data were collected from powder samples on a PANalytical X'Pert powder instrument using CuKα radiation (1.54 Å) operated at 40 kV with 30 mA current. XRD data were recorded over a range of 10–80° with a step time and size of 150 s and 0.02, respectively. BET surface area of the milled samples were measured using Quantachrome Autosorb. The morphology of the milled samples was examined by scanning electron microscopy (SEM), using a Hitachi S4500 Zeiss Supra 55VP microscope operated at 3 to 10 kV. Transmission electron microscopy (TEM) investigations were performed using a JEOL JEM 2100F instrument.

For electrochemical measurements, the anode electrode was prepared by making a homogenous slurry of active material, conductive carbon additive (Super P Li™) and binder (CMC) with a weight ratio of 80% : 10% : 10% in water solvent. The slurry was then uniformly applied onto clean pieces of copper (Cu) foil with an area of 1 × 1 cm^2^. The coated Cu foil was dried naturally for 10 min then in a vacuum oven at 90 °C overnight. The loading of the electrode was around 1–1.5 mg cm^−2^. The dried electrodes were then pressed with 5 tons of load to enhance the contact between material and copper foil. The electrochemical cells (CR2032 coin type cell) were assembled in argon-filled glove box (<0.1 ppm O_2_) with working electrode (active materials coated onto Cu foil), polypropylene film as a separator, and Li metal foil as counter electrode. 1 M LiPF_6_, a mixture of ethylene carbonate (EC)/dimethyl carbonate (DMC)/diethyl carbonate (DEC) (1 : 1 : 1) used as electrolyte. The cells were galvanostatically discharged–charged at 25 °C in the voltage range of 0.01–3.0 V at 250 mA g^−1^ current using a LAND battery testing system (Wuhan LAND Electronics Ltd., China). Cyclic voltammograms (CV) were recorded using an Ivium electrochemical workstation at a scan rate of 0.05 mV s^−1^.

## Results and discussion

**Fig. 2:**
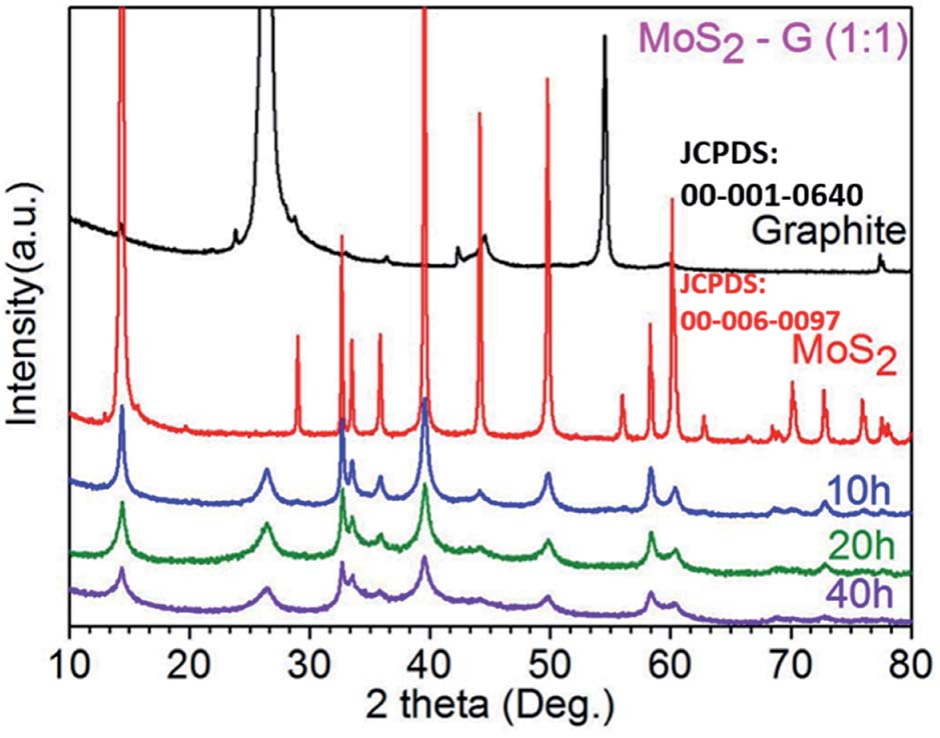
XRD patterns of commercial graphite, commercial MoS_2_ and ball milled MoS_2_-G (10 h), MoS_2_-G (20 h), and MoS_2_-G (40 h) samples.

XRD patterns of the commercial graphite, commercial MoS_2_, and ball-milled three samples of MoS_2_-G (10 h), MoS_2_-G (20 h), and MoS_2_-G (40 h) are shown in Fig. 2. The ball-milled samples show an XRD pattern comprising two phases of graphite (JCPDS: 00-001-0640) and MoS_2_ (JCPDS: 00-006-0097). No peaks of any other phases or impurities were detected within the resolution of the measurement, demonstrating that pure MoS_2_/graphene hybrid can be obtained using ball milling technique.^8–11^ At the same time, the diffraction peaks of the graphite and MoS_2_ phases have a low intensity with respect to the noise level and are significantly broadened, highlighting a small crystallite size of the graphite and MoS_2_. As the milling time increases, the peaks of both commercial graphite and MoS_2_ become wider and weak, indicating the exfoliation of particles to nanosheets.^8^

**Fig. 3:**
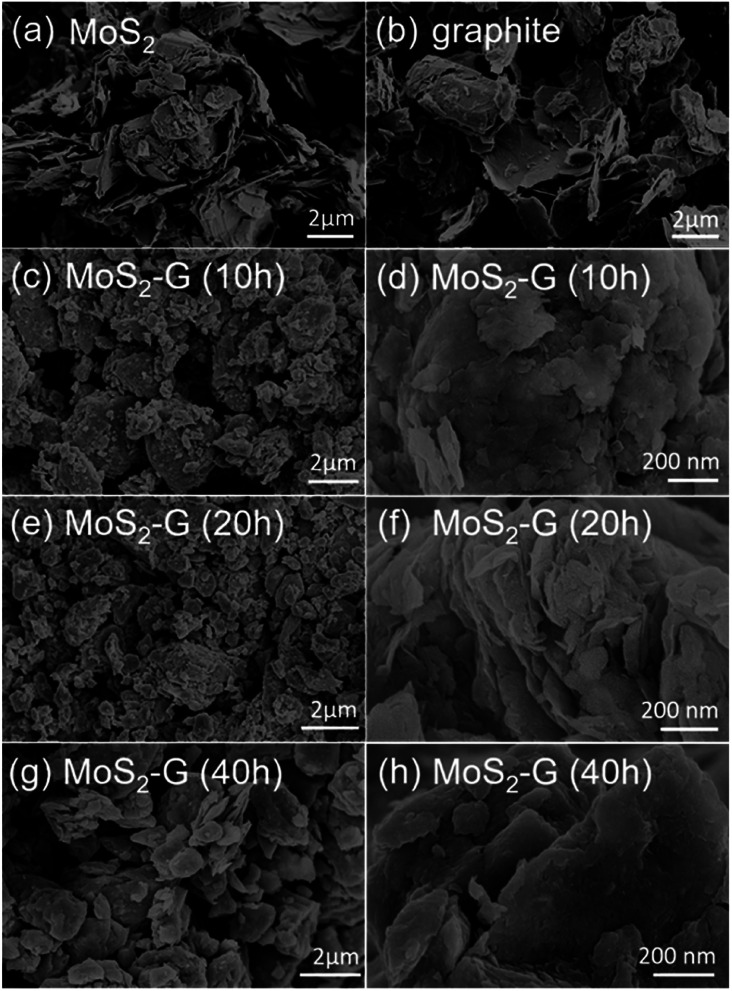
SEM images: (a) commercial MoS_2_, (b) commercial graphite, and (c–h) MoS_2_/graphene hybrid nanosheets of (c and d) MoS_2_-G (10 h), (e and f) MoS_2_-G (20 h), and (g and h) MoS_2_-G (40 h).

Fig. 3 demonstrates SEM images of the pristine MoS_2_ and graphite, and ball milled MoS_2_/graphene hybrid. A typical low magnification SEM images of the pristine MoS_2_ (Fig. 3(a)) and graphite (Fig. 3(b)) reveal that pristine MoS_2_ and graphite consists of numerous large flakes or sheets like morphology. A significant change in morphology is observed between pristine MoS_2_ and graphite, and MoS_2_/graphene hybrids. During ball milling, large flakes of both pristine MoS_2_ and graphite are reduced to small flakes and simultaneously mixed them randomly and form 2D MoS_2_/graphene hybrid nanosheets. A typical morphology of MoS_2_/graphene hybrids is depicted in Fig. 3(c–h). It is clearly observed that MoS_2_ and graphite components are compacted into dense aggregates, which is prospectively beneficial for gaining an attractive capacity and for improvement of packing of the active material in a practical battery electrode. However, the duration of ball milling has an impact in the surface area of the hybrids. The specific BET surface areas of the hybrids were measured by the N_2_ adsorption/desorption method. The MoS_2_-G (20 h) hybrid shows the height surface area of 6.0 m^2^ g^−1^, whereas it was 2.0 m^2^ g^−1^ for MoS_2_-G (10 h) and 4.0 m^2^ g^−1^ for MoS_2_-G (40 h) hybrids. The result of BET surface areas is well consistence with morphology of the hybrids. It is visualised that 10 h and 40 h ball milled samples exhibit denser aggregates than that of 20 h hybrid (compare Fig. 3(d, f and h)). The relatively large specific surface area offers large material/electrolyte contact area, promote electrolyte ion diffusion to the active sites with less resistance^12^ and tolerate the volume change.^13^ Hence, it is anticipated that MoS_2_-G (20 h) hybrid may deliver a high reversible capacity with a stable cycle life as compared to MoS_2_-G (10 h) and MoS_2_-G (40 h) hybrids.

**Fig. 4:**
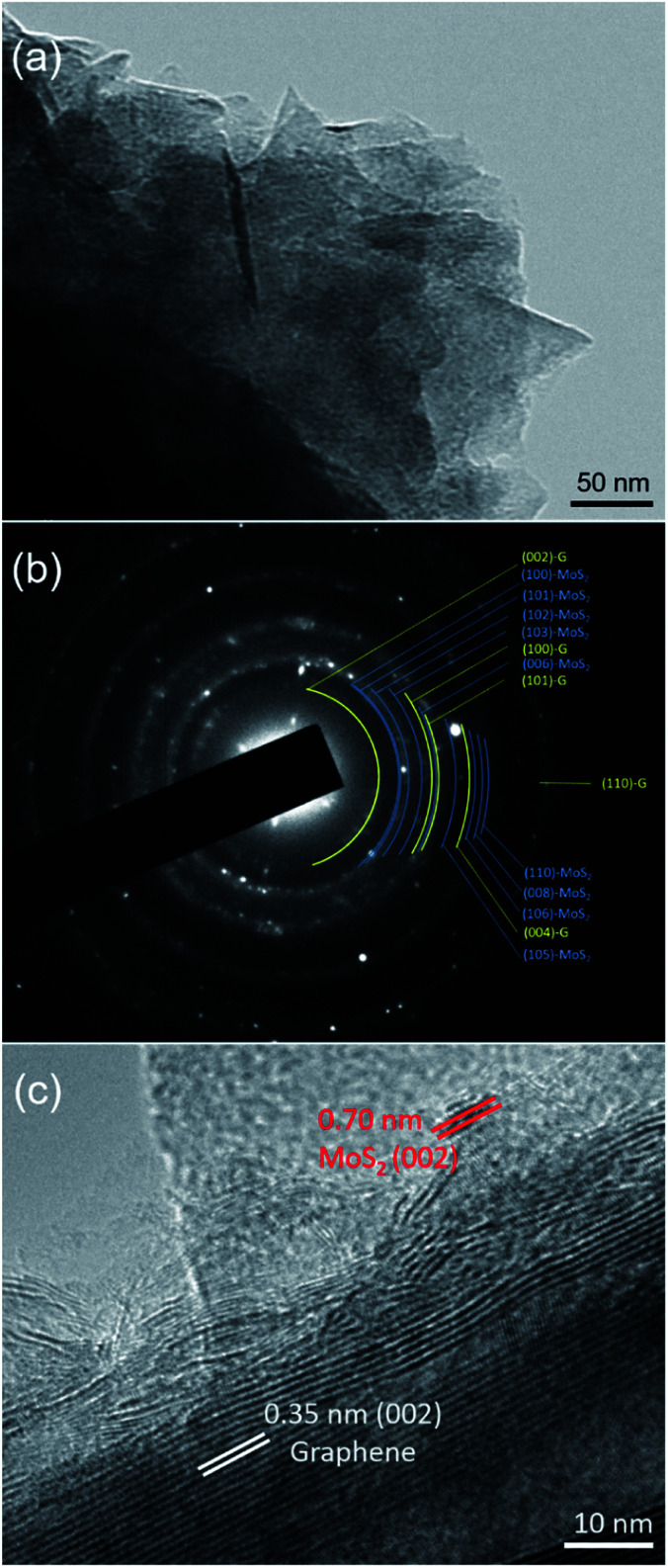
TEM investigation of the MoS_2_-G (20 h) sample: (a) a bright-field TEM image, (b) corresponding SAED pattern, and (c) HRTEM image of the sample.

TEM examination was further carried out to evaluate the structural analysis of the MoS_2_-G (20 h) sample. Bright-field imaging of the sample reveals small flake-like dense morphology (Fig. 4(a)). The corresponding selected area electron diffraction (SAED) pattern of the sample is also depicted in the Fig. 4(b). The SAED pattern consists of both components of MoS_2_ and graphene that can be referenced to the crystallographic directions of (100), (102), (103), and (104) for MoS_2_ and (002), (100), and (101) for graphene, respectively. Fig. 4(c) represents HRTEM image of the sample. The *in situ* generated graphenes are composed of few-layers with a *d* spacing of approximately 0.35 nm, where nanocrystal of MoS_2_ is also clearly visible with a *d* spacing of approximately 0.70 nm (002).

**Fig. 5:**
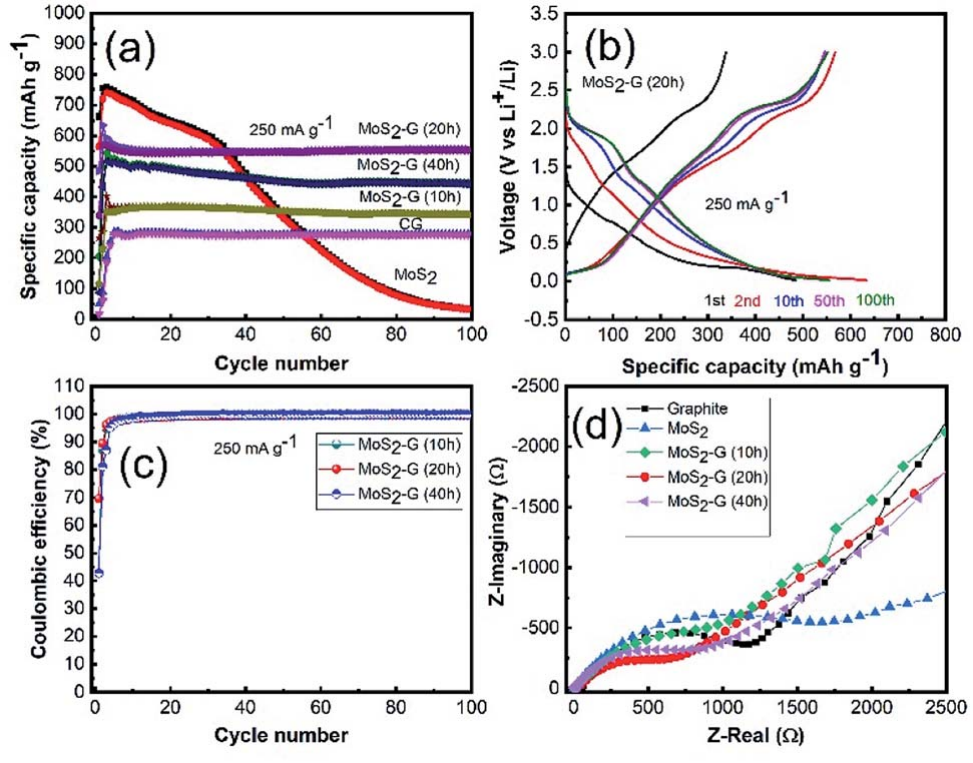
Electrochemical performances of the MoS_2_-G hybrid electrodes: (a) cycling stability up to 100 cycles at a current density of 250 mA g^−1^, (b) galvanostatic discharge/charge voltage profiles of the MoS_2_-G (20 h) electrode, (c) coulombic efficiency of the MoS_2_-G hybrid electrodes, and (d) electrochemical impedance spectra of the fresh cells.

The electrochemical performance of the MoS_2_/graphene hybrid nanosheets was examined using CR 2032 coin-type cells in the voltage range of 0.01–3.0 V. Fig. 5(a) demonstrates the comparison of cycling performance of all electrodes in an identical testing condition. The incorporation of graphene into MoS_2_ has an extraordinary effect on its electrochemical performance. All hybrid electrodes of MoS_2_/G exhibit excellent cycling stability with high reversible capacities of 442 mA h g^−1^ for MoS_2_-G (40 h), 553 mA h g^−1^ for MoS_2_-G (20 h), 342 mA h g^−1^ for MoS_2_-G (10 h) at 250 mA g^−1^ after 100 cycles, respectively. The achieved reversible capacities are almost 85% for MoS_2_-G (40 h), 98% for MoS_2_-G (20 h), and 97% for MoS_2_-G (10 h) electrodes after 100 cycles with respect to the 3^rd^ cycle reversible capacities, respectively. A significant capacity fading is observed for the MoS_2_ electrode with a capacity retention of 37 mA h g^−1^ at 250 mA g^−1^ after 100 cycles. Such a huge improvement in cycling performance of the hybrid electrodes, particularly cycling stability, suggests that the incorporation of graphene into MoS_2_ crystals not only tremendously increases electronic conductivity (both interparticle and intra-particle conductivity) but also improves the ionic conductivities of the hybrid electrodes.^14^ However, it is interesting to note that ball milling duration in the hybrid preparation plays a critical role in their electrochemical performance. Clearly, the MoS_2_-G (20 h) electrode shows better electrochemical performance (in terms of capacity and stability) than that of MoS_2_-G (10 h) and MoS_2_-G (40 h) electrodes, which is related to the high surface area as well as structural integrity of the MoS_2_-G (20 h) electrode.

Corresponding galvanostatic charge–discharge of the MoS_2_-G (20 h) electrode is depicted in Fig. 5(b). Fig. 5(b) shows the first, 2^nd^, 10^th^, 50^th^ and 100^th^ cycle discharge–charge curves of the electrode obtained at 250 mA g^−1^ within the voltage range of 0.01–3.0 V. When the electrode is discharged to 0.01 V (lithiation) and charged to 3.0 V (de-lithiation), both voltage profile exhibits typical plateaus which are consistence with the CV curves. The MoS_2_-G (20 h) electrode also delivers commendable initial coulombic efficiency. At the first cycle, the MoS_2_-G (20 h) electrode exhibits discharge/charge capacities of 485/338 mA h g^−1^, corresponding to the initial coulombic efficiency of ∼70% (Fig. 5(c)). The coulombic efficiency of more than 99% after the first few cycles indicates high reversibility of the composite electrodes, leading to a stable cycling performance. The electrochemical impedance spectroscopy (EIS) of the electrodes was performed in the fresh cells at OCP (open circuit potential) state. Typical Nyquist plots recorded for all electrodes are presented in Fig. 5(d). All plots display one compressed semicircle in the high to medium frequency region and a sloped line in the low frequency region. The diameter of each semicircle is related to the charge transfer resistance, the smaller the diameter, the smaller the charge transfer resistance, and the higher the electronic conductivity.^15,16^ A comparison of the diameters of the semicircles indicates that the impedance of the MoS_2_/graphene hybrid electrodes is significantly lower than that of the graphite and MoS_2_ electrodes, respectively. The incorporation of graphene into MoS_2_ particles under ball milling significantly enhances the conductivity of the MoS_2_/graphene hybrid electrodes, leading to much easier charge transfer at the electrode/electrolyte interface and consequently decreases the overall internal resistance of the cells.

**Fig. 6:**
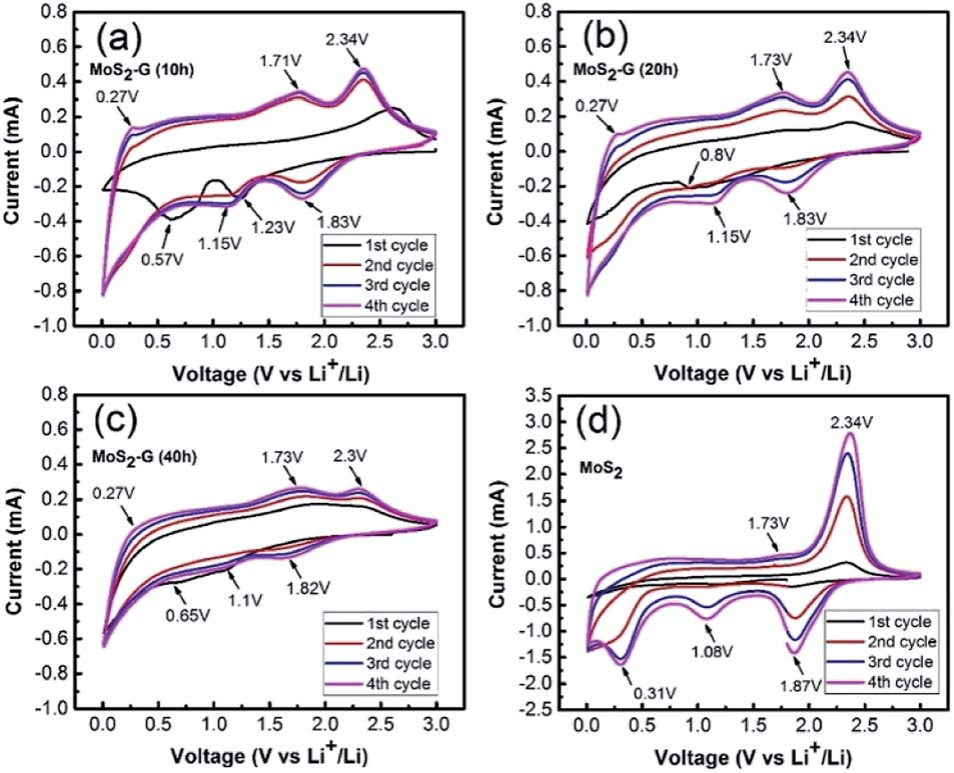
Cyclic voltammograms measured at a scan rate of 0.05 mV s^−1^ in the voltage range 0.01–3.0 V: (a) MoS_2_-G (10 h), (b) MoS_2_-G (20 h), (c) MoS_2_-G (40 h), and (d) MoS_2_.

Fig. 6 shows the cyclic voltammograms of the MoS_2_-G (10 h), MoS_2_-G (20 h), MoS_2_-G (40 h), and MoS_2_ electrodes measured at a scan rate of 0.05 mV s^−1^ in the voltage range 0.01–3.0 V. During the first discharge, two cathodic peaks appear between 1.0–1.25 V and 0.5–0.9 V for the MoS_2_-G electrodes, which can be attributed to the conversion reaction of MoS_2_ + 4Li^+^ → Mo + 2Li_2_S and the change in the coordination of Mo with six S atoms from trigonal prism to octahedron by inserting Li ions into the MoS_2_ layers (*i.e.*, formation of Li_*x*_MoS_2_) (1.0–1.25 V).^2^ It is observed that the cathodic peaks appeared between 0.5–0.9 V comprises broad peak, which should be correlated with the formation of a SEI (solid electrolyte interface) film on the surface of the electrodes.^17^ In the subsequent discharge process, the cathodic peaks are quite different from the first discharge and peaks are shifted to higher voltages and two new broad peaks appeared at 1.0 and 1.8 V, corresponding to the voltage plateau with the formation of Li_2_S and the association of Li ions with Mo.^18^ In the anodic scans, the electrodes display two peaks at 1.7 and 2.3 V, which are corresponding to the oxidation of Mo metal to MoS_2_ (1.7 V), while the pronounced oxidation peak at 2.3 V is due to the formation of sulfur.^19,20^ The MoS_2_ bulk electrode (Fig. 6(d)) shows three sharp cathodic peaks at 1.87, 1.08, and 0.31 V. In the anodic process, the MoS_2_ electrode exhibits two peaks at 1.7 V and 2.3 V, corresponding to the oxidation of Mo metal to MoS_2_ and formation sulfur. However, a pair of cathodic/anodic peak at around 0.19/0.27 V is visualised for the MoS_2_-G electrodes, which is assigned to the insertion/extraction of Li ions into the layer of graphene.^2^

The obtained preliminary results indicate that the structure of 2D MoS_2_/graphene electrode is beneficial to the full infiltration of the electrolyte, reducing the migrating distance of Li ions, facilitating Li-ions exchange across the heterointerface by maintaining a large effective contact area between the active materials, conductive additives, and the electrolyte. Most importantly, the 2D MoS_2_/graphene hybrid nanosheets structure is capable of tolerating volume variation of the electrodes during Li-ions extraction/insertion, enhancing Li-ions storage kinetics and structural stability. The obtained electrochemical performance of the 2D MoS_2_/graphene hybrid electrode is promising and comparable with those of the reported results^21–29^ as summarised in Table S1 (ESI).† It is expected that a more robust electrode architecture of MoS_2_/graphene hybrid nanosheets prepared by controlled parameters could be used as an effective negative electrode for more sustainable, cost-effective Na-ion and K-ion batteries and this is to be demonstrated in our future work.

## Conclusions

To prevent the long-standing issue of poor cycling stability of MoS_2_ anode in lithium-ion batteries, a hybrid of nanostructured MoS_2_ with graphene is proposed. A simple and scalable ball milling method is developed to produce MoS_2_/graphene hybrid nanosheets *via in situ* mechanical peeling of bulk graphite and simultaneously disperse them among MoS_2_ nanosheets. The MoS_2_/graphene hybrid nanosheets effectively lead to significant improvements in electronic conductivity, structural stability, and ion diffusion, which in turn, results in excellent cycling stability is a significant advance in the field.

## Conflicts of interest

There are no conflicts to declare.

## Supplementary Material

RA-010-D0RA01503B-s001
