# Supplementary material for: In situ production of a two-dimensional molybdenum disulfide/graphene hybrid nanosheet anode for lithium-ion batteries
Source: RSC Adv. 2020 Apr 14;10(22):12754–8. doi: 10.1039/d0ra01503b (PMC9051260; doi:10.1039/d0ra01503b)
Supplement: RA-010-D0RA01503B-s001 [file RA-010-D0RA01503B-s001.pdf]

Electronic Supplementary Material (ESI) for RSC Advances.
This journal is © The Royal Society of Chemistry 2020

Electronic Supplementary Information (ESI)

In-situ production of two-dimensional molybdenum disulfide/graphene hybrid nanosheets anode for lithium-ion batteries

Srikanth Mateti,[a] Md Mokhlesur Rahman,*[a] Pavel Cizek[a] and Ying Chen*[a]

[a]Institute for Frontier Materials, Deakin University, Geelong Campus, Waurn Ponds, 3216, Victoria, Australia.

Corresponding authors:

E-mail: m.rahman@deakin.edu.au; ian.chen@deakin.edu.au

Table S1. Electrochemical performances of the reported MoS_2-based anode materials for lithium-ion batteries.

Materials	Cycling performance		Method	Ref.
	Capacity ($mAh\ g^{-1}$)	Current ($mA\ g^{-1}$)		
2D MoS_2/graphene hybrid	553	250	Ball mill	This work
MoS_2/graphene heterostructure	786	100	Hydrothermal	21
MoS_2-rGO composites	908	100	Microwave annealing	22
MoS_2-RGO composites	896	50	Supercritical methanol	23
Layer-by-layer MoS_2/rGO hybrids	940	100	Intercalation exfoliation	24
MoS_2/graphene hybrids	800	100	heat-treatment	25
MoS_2/graphene hybrid nanosheets	902	100	Hydrothermal	26
Monodispersed MoS_2 hierarchical architecture	839	100	Hydrothermal	27
MoS_2/graphene nanocomposite	767	100	Hydrothermal	28
Hierarchical MoS_2/G composite	1077	100	Hydrothermal	29

References

21 H. Liu, X. Chen, L. Deng, X. Su, K. Guo and Z. Zhu, *Electrochim. Acta,* 2016, **206**, 184.

22 D.H. Young, C. Jo, J.Y. Kim, J. Lee and J.S. Lee, *J. Power Sources,* 2015, **295**, 228.

23 M. Choi, S.K. Koppala, D. Yoon, J. Hwang, S.M. Kim and J. Kim, *J. Power Sources*, 2016, **309**, 202.

24 Y. Jing, E.O. Ortiz-Quiles, C.R. Cabrera, Z. Chen and Z. Zhou, *Electrochim. Acta*, 2014, **147**, 392.

25 S. Srivastava, B. Kartick, S. Choudhury and M. Stamm, *Mater. Chem. Phys.,* 2016, **183**, 383.

26 X. Zhang, Q. Zhang, Y. Sun, P. Zhang, X. Gao, W. Zhang and J. Guo, Electrochim. Acta, 2016, **189**, 224.

27 L. Xu, L. Ma, T. Rujirala, B. Liu, J. Zhang and W. Zhang, *Nanotechnology*, 2019, **30**, 415402.

28 L. Chen, F. Chen, N. Tronganh, M. Lu and Y. Jiang, *J. Mater. Res.,* 2016, **31**, 3151.

29 Y. Teng, H. Zhao, Z. Zhang, Z. Li, Q. Xia, Y. Zhang, L. Zhao, X. Du, Z. Du, P. Lv and K. Świerczek, *ACS Nano*, 2016, **10**, 8526.
